# Cerebrovascular burden and neurodegeneration linked to 15-year odor identification decline in older adults

**DOI:** 10.3389/fnagi.2025.1539508

**Published:** 2025-03-24

**Authors:** Javier Oltra, Grégoria Kalpouzos, Ingrid Ekström, Maria Larsson, Yuanjing Li, Chengxuan Qiu, Erika J. Laukka

**Affiliations:** ^1^Aging Research Center, Department of Neurobiology, Care Sciences and Society, Karolinska Institutet and Stockholm University, Stockholm, Sweden; ^2^Gösta Ekman Laboratory, Department of Psychology, Stockholm University, Stockholm, Sweden; ^3^Stockholm Gerontology Research Center, Stockholm, Sweden

**Keywords:** olfaction, microvascular lesions, brain atrophy, population-based study, aging, dementia

## Abstract

**Background:**

The mechanisms underlying olfactory decline in aging need further investigation. Noticeably, the longitudinal relationship of biological markers with olfaction remains underexplored. We investigated whether baseline levels and progression of microvascular lesions and brain atrophy are associated with odor identification (OID) decline.

**Methods:**

The association between structural MRI markers and OID decline was examined in participants from the SNAC-K MRI study who were free from dementia at baseline (*n* = 401, mean age = 70.2 years, 60% females). OID was repeatedly assessed over 15 years. Presence of lacunes, white matter hyperintensities (WMH), perivascular spaces (PVS), and lateral ventricular, hippocampal, amygdalar, and total gray matter (GM) volumes were assessed up to 6 years, concurrent with the first 6 years of olfactory assessments.

**Results:**

Higher PVS count and lower hippocampal and GM volumes at baseline were associated with accelerated OID decline (*p_FWE_* < 0.05). Longitudinally (*n* = 225), presence of lacunes at follow-up, faster WMH volume and PVS count increases, faster lateral ventricular enlargement, and faster hippocampal, amygdalar, and GM atrophy were associated with accelerated OID decline (*p*_FWE_ < 0.05).

**Conclusion:**

Olfactory decline is related to both increased cerebrovascular burden and accelerated brain atrophy over time.

## 1 Introduction

Odor identification (OID) dysfunction, characterized by an impairment of the ability to correctly identify common odors, is a well-established early marker of cognitive decline (Dintica et al., 2019; Tian et al., 2023), mild cognitive impairment (MCI) (Roberts et al., 2016; Dong et al., 2023), and all-type dementia (Adams et al., 2018; Laukka et al., 2023) and Alzheimer’s disease (AD) dementia (Murphy, 2019). Though OID starts to decline in the fifth decade of life (Zhang and Wang, 2017), recent studies have shown that a significantly accelerated decline predicts progression to MCI (Guo et al., 2023; Pacyna et al., 2023), dementia (Pacyna et al., 2023), and the transition between them, even when the performance falls within the normal range (Guo et al., 2023).

Despite increasing interest in the topic, some questions remain regarding the mechanisms underlying OID decline in aging and the observed associations with dementia and cognitive impairment. Besides degeneration of the peripheral olfactory system, changes in the central nervous system have been hypothesized to play a crucial role in OID decline (Bothwell et al., 2023; Parvand and Rankin, 2020), especially since OID ability has a significant cognitive component (Jobin et al., 2023b; Larsson et al., 2016). In this context, brain atrophy has emerged as one of the potential leading contributing factors of OID decline in aging. Previous population-based studies of cognitively normal (CN) elderly using structural magnetic resonance imaging (sMRI) showed that lower gray matter (GM) volume (Devanand et al., 2010; Diez et al., 2024; Dintica et al., 2019; Growdon et al., 2015; Kamath et al., 2022; Segura et al., 2013; Seubert et al., 2020; Tan et al., 2022; Vassilaki et al., 2017), thickness (Growdon et al., 2015), and integrity (Shrestha et al., 2023), as well as a faster volume decrease (Tian et al., 2023) particularly in medial temporal lobe (MTL) structures (e.g., hippocampi, amygdalae, entorhinal cortex) were associated with poorer OID. Furthermore, a recent study reported an association between smaller GM volume in olfactory, frontoparietal, and temporal regions with accelerated OID decline (Pacyna et al., 2023). Another study examined the longitudinal association between GM measures and OID exploring hippocampal subfield volumes, reporting an association between faster hippocampal tail, subiculum, CA4, and dentate gyrus decreases and accelerated OID decline in older adults (Menelaou et al., 2022).

Generally, there is a consistent link between GM measures of the MTL and OID in aging—for a systematic review, see Bothwell et al., 2023. MTL atrophy may also affect memory processing (Bettio et al., 2017), potentially impacting a key cognitive component of OID ability. Beta-amyloid deposition may be one potential process linking brain atrophy and OID decline in aging, as suggested by meta-analytic evidence indicating an association between amyloid PET and poorer OID performance (Tu et al., 2020).

So far, research in this area has primarily focused on markers of neurodegeneration. Notably, previous studies have reported associations of olfactory dysfunctions with cerebrovascular (Murphy et al., 2002; Omori and Okutani, 2020; Palmquist et al., 2020) and cardiovascular diseases (Palmquist et al., 2020; Roh et al., 2021; Schubert et al., 2015; Seubert et al., 2017) and related risk factors (Dong et al., 2021; Palmquist et al., 2020; Roh et al., 2021; Siegel et al., 2019). In this regard, a previously proposed cerebrovascular mechanism hypothesizes that a reduced blood flow due to atherosclerosis of the supplying blood vessels could lead to hypoxia affecting key olfactory processing areas (Schubert et al., 2015; Siegel et al., 2019). In addition, it is noteworthy that cerebrovascular co-pathologies are also common in AD and Lewy body diseases [i.e., Parkinson’s disease (PD) and dementia with Lewy bodies] (Attems and Jellinger, 2014; Hijazi et al., 2022), which have a high prevalence of olfactory dysfunctions (Bagherieh et al., 2023; Silva et al., 2018). Regarding brain sMRI markers of microvascular lesions, previous population-based studies in CN older adults showed mixed cross-sectional findings on the link between white matter hyperintensities (WMH) burden and OID, pointing to a negative association (Dong et al., 2023) or no relation (Buchanan et al., 2020; Devanand et al., 2010).

Overall, the evidence supports a link between brain atrophy and OID dysfunction. However, previous studies generally lacked markers of cerebral microvascular pathology, limiting them to WMH when included. Moreover, most studies were either based on cross-sectional brain data or on a single time-point olfactory assessment. Thus, the longitudinal association of microvascular burden and brain atrophy accumulation with OID decline remains largely unexplored. The lack of studies including white matter (WM) lesions, which may be extensible to other types of microvascular lesions, and longitudinal approaches has been highlighted as two of the main research gaps in the field (Bothwell et al., 2023). A deeper understanding of brain-olfaction relations is pivotal to determining which early brain changes are potentially related to olfactory deterioration in aging and predementia stages.

To advance knowledge about the brain correlates of olfactory deficits in aging, we aimed to examine whether (1) various sMRI markers of brain atrophy and microvascular lesions and (2) their progression are associated with OID decline in a population-based cohort of older adults.

## 2 Methods

### 2.1 Participants

This population-based cohort study comprised participants from the MRI subsample of the Swedish National Study on Aging and Care in Kungsholmen (SNAC-K), a multidisciplinary study of aging and health (Lagergren et al., 2004). SNAC-K includes individuals aged 60 years or older living at home or in an institution in the Kungsholmen district of central Stockholm. The sampling design included three younger cohorts (aged 60 to 72 years, with a 6-year interval) and eight older cohorts (aged 78 to 99+ years, with a 3-year interval). This design was tailored to capture the aging process, including the work-retirement transaction, and the follow-up intervals were shorter for individuals over 80 intending to capture potentially faster changes in older age groups. Participants are called back when they reach the age of the next cohort (i.e., every 3 or 6 years). Each data collection wave includes a nurse interview, a medical examination, and a neuropsychological assessment.

From a pool of 3,363 eligible participants at baseline (2001–2003), 555 non-disabled and noninstitutionalized participants, able to attend the visit and not living in a nursing home, underwent a baseline brain MRI scan (Wang et al., 2014), with follow-up scans performed at 3 and 6 years. Out of these 555 participants, we excluded 154 participants due to incomplete MRI data, suboptimal quality of images, presence of brain infarcts brain tumors, or arachnoid cysts (*n* = 102), missing OID score (*n* = 44), having a diagnosis of dementia (*n* = 1), PD (*n* = 3), Guillain-Barré syndrome (*n* = 1), and epilepsy (*n* = 1), and aged over 90 years (*n* = 2), leaving 401 free of dementia participants for analyses regarding the association of sMRI markers at baseline with OID decline (*analytical sample 1*). Of those 401 participants, follow-up sMRI data were available in 225 persons, constituting the sample for analyses regarding the progression of sMRI markers and OID decline (*analytical sample 2*). We excluded 35 participants due to incomplete MRI data, suboptimal quality of images, presence of brain infarcts, brain tumors, or arachnoid cysts, and 141 participants due to missing MRI data. Supplementary Figure 1 shows the flowchart of study participants, including applied exclusion criteria.

In the present study, we used follow-up sMRI data up to 6 years and olfactory data up to 15 years from baseline (where the first 6 years were concurrent to the MRI examination). Of all participants in this study, 338 out of 401 (*analytical sample 1*) and 220 out of 225 (*analytical sample 2*) had available OID follow-up data.

### 2.2 Procedures

#### 2.2.1 Odor identification assessment

OID was assessed at baseline and follow-ups with the Sniffin’ Sticks, a well-established test with normative standard scores and high test–retest reliability, following the procedure described in Croy et al. (2015). The test administration in SNAC-K is explained in detail in previous publications (Ekström et al., 2020; Larsson et al., 2016). Briefly, the evaluator asked participants to identify 16 common household odors presented individually in felt-tip pens. First, they tried to identify each odor freely. If they failed the free identification, the evaluator provided them with four written alternatives (one target and three foils) and instructed them to select the one that best matched the odor (i.e., cued identification). Participants received a score of 0 for an item when incapable of correctly identifying it. On the rare occasion when information for an item was missing (e.g., due to an administration mistake or allergy), participants received a score of 0.25 (i.e., equivalent to chance level). In this study, we used the OID score representing the number of correctly identified odors with either free or cued identification (range 0 to 16).

For further sensitivity analyses, we classified participants scoring ≤6 in the Sniffin’ Sticks test at baseline as anosmics, based on normative data (Kobal et al., 2000; Hummel et al., 2007; Oleszkiewicz et al., 2019).

#### 2.2.2 Cognitive assessment and dementia diagnosis

The neuropsychological assessment, in which the OID test was included, addressed semantic memory (vocabulary and general knowledge), episodic memory (word free recall and recognition), attention and executive function (Trail Making Test), language (letter and category fluency), visuospatial abilities (mental rotations), and perceptual speed (pattern comparison and digit cancellation), as detailed elsewhere (Pantzar et al., 2014), see details in Supplementary methods. We specifically selected semantic memory performance as a possible confounding factor to account for the cognitive component in the olfactory assessment, particularly for the declarative memory component (Larsson et al., 2016; Jobin et al., 2023b). It was assessed using the SRB:1, a 30-item multiple-choice Swedish vocabulary test from the Synonym Reasoning Blocks, consisting of the identification of synonyms within a 7-min time frame (range 0 to 30) (Dureman, 1960; Nilsson et al., 1997). The test has a similar structure to the Sniffin’ Sticks test. The participant is instructed to select a synonym of the written stimulus word from five written alternatives (one target and four foils). We used mean-centered semantic memory performance based on *analytical sample 1* as a covariate in further analyses.

Dementia diagnosis at baseline and follow-up, used for sample selection and to exclude participants in sensitivity analyses, followed the *Diagnostic and Statistical Manual of Mental Disorders, 4th edition* (*DSM-IV*) criteria. In the SNAC-K study, the participants received a preliminary diagnosis after undergoing a medical examination, including a neurological examination and assessment of clinical history, drug use, and cognitive functioning. A second physician made a second independent diagnosis based on data documented from the medical examination. In case of disagreement, the final diagnosis was made by a senior neurologist. The cognitive tests used for dementia diagnoses included the MMSE (Folstein et al., 1975), the Clock test (Manos and Wu, 1994), and items regarding memory, executive functioning, problem-solving, orientation, and interpretation of proverbs. Additional dementia cases among participants who died between two assessments and developed dementia after their last examination were identified using the Swedish National Cause of Death Register, clinical charts, and medical records. In total, 57 participants were diagnosed with dementia during the 15-year follow-up (mean time to diagnosis = 9.55 years, standard deviation, *SD* = 3.86), the majority of which were classified as AD dementia or mixed dementia. Moreover, 1 participant was clinically diagnosed with PD (time to diagnosis = 5.71 years).

### 2.3 MRI acquisition

The participants underwent baseline and follow-up brain MRI examinations on a Philips Intera 1.5 T MRI scanner (The Netherlands). The core protocol included a 3D axial magnetization-prepared rapid gradient-echo (MP-RAGE) T1-weighted sequence (resolution: 0.94 × 0.94 × 1.5 mm; no gap; repetition time, TR = 15 ms; echo-time, TE = 7 ms; flip angle, FA = 15°), a proton-density/T2-weighted sequence (resolution: 0.98 × 0.98 × 3 mm; no gap; TR = 3,995 ms; TE = 18/90 ms; echo-train length, ETL = 6; FA = 90°), and a fluid-attenuated inversion recovery (FLAIR) sequence (resolution: 0.90 × 0.90 × 6 mm; gap between slices: 1 mm; TR = 6,000 ms; TE = 100 ms; inversion time, TI = 1900 ms; ETL = 21; FA = 90°).

### 2.4 Structural MRI markers

Lateral ventricular, hippocampal, and total GM volumes were considered markers for brain atrophy. Hippocampal and amygdalar volumes were selected as markers related to AD neuropathology and early AD-related neurodegeneration (Kotrotsou et al., 2015; Planche et al., 2022), being structures consistently associated with OID in aging, MCI, and AD (Bothwell et al., 2023). Hippocampi and amygdalae are part of the secondary and primary olfactory cortices, receiving projections from the primary olfactory cortex and being one synapse away from the olfactory bulb, respectively (Cersosimo et al., 2021). Lateral ventricular volume, which is a marker of global GM and WM atrophy based on its neuropathologic correlates (Erten-Lyons et al., 2013), and total GM volume were selected to examine broad neurodegeneration since OID is associated with lower GM measures and WM metrics encompassing other brain regions apart from MTL structures (Bothwell et al., 2023; Felix et al., 2021; Shao et al., 2021). The lateral ventricular volume was measured using ALVIN’s algorithm, hippocampal and amygdalar volumes using automated segmentation from FreeSurfer version 5.1, and total GM volume using SPM12 in MATLAB R2012b as detailed in previous publications (Gerritsen et al., 2015; Wang et al., 2018).

As markers indicating cerebral microvascular lesions, we assessed lacunes, WMH, and enlarged perivascular spaces (PVS), since they are assumed to reflect predominantly microvascular damage in the brain (Jagust et al., 2008; Young et al., 2008). A trained rater (Y.L.) supervised by a senior neuroimaging analyst (G.K.) visually evaluated and counted lacunes and PVS as described elsewhere (Li et al., 2022). A senior neuroimaging analyst (G.K.) manually drew global WMH volume on FLAIR images as previously described (Köhncke et al., 2016).

All sMRI data was visually inspected and quality checked by a senior neuroimaging analyst (G.K.).

Furthermore, we computed estimated total intracranial volume (eTIV) by summing GM, WM, and cerebrospinal fluid volumes using SPM12 (Wang et al., 2018). Next, all volumetric variables were adjusted by eTIV using a residual approach by implementing a linear regression between the volumetric measures and baseline eTIV to predict the eTIV-adjusted volumes based on *analytical sample 1* (Jack et al., 1989). Due to skewed distribution, we log-transformed WMH volume by using the formula *ln(x + 1)* after setting the lowest observation at 0 to avoid negative values, by summing the minimum value multiplied by −1 to all observations. Next, we z-transformed all volumetric measures and the global PVS count based on the baseline mean and standard deviation of *analytical sample 1*.

We computed the progression of sMRI markers for *analytical sample 2*. For this purpose, we estimated the annual average change (AAC; time unit, years) for each participant in volumetric measures and global PVS count as the sum of the fixed coefficient of follow-up time and the random coefficient for that participant using linear mixed-effects models (Li et al., 2022, 2023). The longitudinal presence of lacunes was set as the presence of lacunes at the longest available follow-up, regardless of its presence at baseline, which was used as a covariate in further analyses.

### 2.5 Assessment of covariates

Demographics (age, sex, and education) and lifestyle information (smoking status) were collected during the nurse interview. Education was measured as years of formal schooling and smoking status was dichotomized as never/former or current smoking. We used mean-centered age and education based on *analytical sample 1* in further analyses.

Reduced sleep quality/duration of sleep at baseline was assessed with an item from the Comprehensive Psychopathological Rating Scale (CPRS) (Asberg and Schalling, 1979; Overton et al., 2024). The item measured “a subjective feeling of reduced duration or depth of sleep compared with the subject’s own normal pattern.” The answer alternatives were: “I sleep as normal” (0–1); “moderate difficulties in initiating sleep, or shorter, lighter, or disturbed sleep” (2–3); “reduced sleep with at least 2 h per night or early awakenings without external influence” (4–5); and “less than 2–3 h sleep per night” (6). The responses were dichotomized as normal sleep (0–1) or reduced sleep (2–6).

Information on vascular risk factors and cardiovascular health was collected via the nurse interview, medical examination, and National Patient Register (Shang et al., 2020). *APOE* genotyping (rs429358, rs7412) was performed using MALDI-TOF analysis on the Sequenom MassARRAY™ platform at the Mutation Analysis Facility, Karolinska Institutet. We dichotomized *APOE* into carriers versus non-carriers of the ε4 allele.

### 2.6 Statistical analyses

We characterized the baseline profile of participants with and without available follow-up sMRI data and compared them using t-tests for continuous variables and chi-square tests for categorical variables.

Linear mixed-effects models were performed using Stata Statistical Software (version 18; StataCorp LLC, College Station, TX). First, we estimated the association of baseline sMRI markers with AAC in OID in *analytical sample 1* to address the question of whether the baseline levels of microvascular lesions and brain atrophy were associated with OID decline. Participant identifier was set as a categorical random effect to account for the repeated measures from each participant. Next, we estimated the association of AAC in individual sMRI markers and the presence of lacunes at follow-up with AAC in OID in *analytical sample 2* to address the question of whether the progression of microvascular lesions and brain atrophy was associated with OID decline.

The models included sMRI variables, follow-up time from baseline (in years), and their interaction. We applied unstructured covariance matrices and used maximum likelihood to impute missing olfactory data during follow-up for all models. For each model, we report *β*-coefficients (95% confidence interval, CI) for the interaction term, as an index of the additional effect of the sMRI variables on AAC in OID (i.e., over and above the annual average OID decline). In all models, we controlled for sex, age in years, and years of formal schooling (i.e., sociodemographic covariates). When examining the association of the AAC in sMRI markers with the AAC in OID, we included baseline sMRI markers as covariates, and the presence of lacunes at baseline when examining incident lacunes at follow-up.

We report the results from three main models. *Model 1* included each sMRI marker individually and sociodemographic covariates, for this basic model, we applied Holm’s Sequential Bonferroni Procedure to control the family-wise error rate (FWER) (Eichstaedt et al., 2013) and reported their unadjusted and adjusted *p* values. *Model 2* included all sMRI markers simultaneously to assess their statistically independent effects, along with sociodemographic covariates. *Model 3* included semantic memory performance and smoking status as extra covariates in *model 2* to control for the cognitive component of the OID task (Jobin et al., 2023b; Larsson et al., 2016) and for a health behavior affecting olfactory function (Ajmani et al., 2017), respectively.

Additionally, we ran the models including the three-way interaction between baseline or longitudinal sMRI variables and follow-up time with sex, age groups (i.e., <78 vs. ≥78 years, young-old age vs. old-old age, based on the sampling design), and *APOE* ε4 genotype variables, which have previously shown a significant association with OID performance (i.e., sex) and decline (i.e., age groups and *APOE* ε4 allele) in the SNAC-K sample (Ekström et al., 2020). If a statistically significant interaction was detected, stratified analyses by sex, age groups, or *APOE* ε4 genotype were further performed to determine its direction and magnitude.

As supplementary analyses, we used linear mixed models to examine whether PVS counts at baseline and longitudinal increases have differential effects on OID decline depending on the affected regions in aging. If a higher global PVS count at baseline or a faster increase in PVS count was associated with accelerated OID decline, we examined regional PVS counts (i.e., lobar, infratentorial, and deep regions), followed by further subdivisions as appropriate (i.e., frontal and parieto-occipital for lobar PVS count; mesencephalon and cerebellum for infratentorial PVS count; basal, ganglia, sub-insular, and hippocampal for deep PVS count). Importantly, the continuous network of PVS known as glymphatic system is suggested to be responsible for clearing waste products from the brain (Iliff et al., 2012; Nedergaard and Goldman, 2020), being especially active during sleep (Xie et al., 2013; Hablitz et al., 2020; Dredla et al., 2023). Poor sleep quality has been previously associated with a glymphatic impairment in aging (Baril et al., 2022; Lysen et al., 2022; Siow et al., 2022), potentially affecting memory performance (Ma et al., 2024). In neurodegenerative diseases, a regional association between PVS and OID has been previously reported in PD (Fang et al., 2020), which is characterized by sleep disturbances from prodromal stages. Thus, we computed analyses both controlling for sociodemographic covariates and additionally controlling for reduced sleep to assess the impact of sleep quality on these associations.

We performed two sets of sensitivity analyses. First, we re-ran all main models after excluding 13 participants with anosmia at baseline. We aimed to verify if including participants displaying a significant olfactory impairment at baseline could affect the results. Second, we re-ran all main models after excluding 58 participants diagnosed with dementia (*n* = 57) or PD (*n* = 1) during the 15-year follow-up period. We aimed to examine whether removing participants with an expected pathological brain aging and olfactory trajectory impacts the associations. Complementary, we compared the characteristics of the anosmic and incident dementia or PD groups with the rest of the sample using *t*-tests or Mann–Whitney *U* tests for continuous variables and chi-square or Fisher’s exact tests for categorical variables, as appropriate. Differences in the AACs of OID and sMRI markers were assessed using linear mixed models testing the two-way interactions between follow-up time and group (e.g., non-anosmic vs. anosmic) adjusting for sociodemographic covariates.

## 3 Results

Descriptive information for the study population can be found in Table 1. The mean age at baseline was 70.2 years (*SD* = 8.7), 59.9% were females, and the average amount of formal schooling was 12.7 years (*SD* = 4.3). When comparing participants with and without follow-up sMRI markers, those without follow-up markers were older at baseline, had fewer years of formal schooling, showed lower semantic memory performance, and displayed higher WMH and lateral ventricular volumes, along with lower hippocampal and total GM volumes (*p* < 0.05, Table 1). Importantly, the two groups did not differ significantly in OID, global cognition, global PVS count, proportion of current smokers and *APOE* ε4 carriers, having hypertension and diabetes mellitus, and presence of lacunes (*p* > 0.05, Table 1). In addition, participants without follow-up markers displayed lower performance in episodic memory, attention and executive function, language, and perceptual speed tests (*p* < 0.05, Supplementary Table 1).

**Table 1:** Baseline characteristics of study participants in the total sample and by availability of sMRI markers at follow-up.

	Total sample	Follow-up sMRI markers		
Characteristics	(*n* = 401)	No (*n* = 176)	Yes (*n* = 225)	*p*
Age (y), *M* (*SD*)	70.2 (8.7)	71.5 (9.1)	69.2 (8.2)	0.007
Age groups				
Young-old (<78), *n* (%)	282 (70.3)	117 (66.5)	165 (73.3)	0.136
Old-old (≥78), *n* (%)	119 (29.7)	59 (33.5)	60 (26.7)	
Female, *n* (%)	240 (59.9)	99 (56.3)	141 (62.7)	0.193
Education (y), *M* (*SD*)	12.7 (4.3)	12.1 (4.2)	13.2 (4.3)	0.008
MMSE score, *M* (*SD*)	29.1 (1.0)	29.0 (1.1)	29.2 (1.0)	0.064
Hypertension, *n* (%)	170 (42.4)	80 (45.5)	91 (40.0)	0.273
Diabetes mellitus, *n* (%)	28 (7.0)	15 (8.5)	13 (5.8)	0.284
Current smoking, *n* (%)	57 (14.2)	28 (15.9)	29 (12.9)	0.390
Reduced sleep, *n* (%)^a^	45 (11.2)	16 (9.3)	29 (13.1)	0.238
*APOE* ε4 allele				
Non carrier, *n* (%)	276 (68.8)	118 (67.0)	158 (70.2)	0.912
Carrier, *n* (%)	113 (28.2)	49 (27.8)	64 (28.4)	
Missing, *n* (%)	12 (3.0)	9 (5.1)	3 (1.3)	
Olfactory assessment follow-up time (y), *M* (*SD*)	10.4 (3.6)	9.0 (3.8)	11.2 (3.2)	<0.001
Olfactory assessments per person (*n*), *M* (*SD*)	2.9 (1.2)	2.3 (1.1)	3.4 (1.0)	<0.001
MRI examination follow-up time (y), *M* (*SD*)	NA	NA	5.4 (0.9)	NA
MRI examinations per person (*n*), *M* (*SD*)	NA	NA	2.1 (0.3)	NA
OID score, *M* (*SD*)	12.1 (2.6)	11.9 (2.7)	12.3 (2.5)	0.151
Semantic memory score, *M* (*SD*)^a^	23.6 (4.3)	23.1 (4.9)	24.0 (3.8)	0.040
eTIV (mL), *M* (*SD*)	1493.4 (154.9)	1500.5 (148.2)	1487.8 (160.1)	0.418
sMRI markers				
Presence of lacunes, *n* (%)	72 (18.0)	35 (19.9)	37 (16.4)	0.373
WMH volume (mL), *M* (*SD*)	7.0 (9.5)	8.2 (10.3)	6.1 (8.8)	0.031
Global PVS count, *M* (*SD*)	82.9 (26.1)	84.4 (28.3)	81.7 (24.1)	0.310
Lateral ventricular volume (mL), *M* (*SD*)	38.1 (16.2)	41.8 (17.7)	35.2 (14.4)	<0.001
Hippocampal volume (mL), *M* (*SD*)	7.5 (0.8)	7.4 (0.8)	7.6 (0.8)	0.002
Amygdalar volumen (mL), *M* (*SD*)	2.7 (0.3)	2.7 (0.3)	2.7 (0.3)	0.110
Total GM volume (mL), *M* (*SD*)	551.2 (52.3)	544.3 (54.7)	556.7 (49.8)	0.018

eTIV, estimated total intracranial volume; GM, gray matter; M, mean; MMSE, Mini Mental State Examination; NA, not applicable; OID, odor identification; PVS, perivascular spaces; SD, standard deviation; sMRI, structural magnetic resonance imaging; WMH, white matter hyperintensities.

a The number of participants with missing values was 8 for reduced sleep, 4 belonging to the group of participants without available sMRI markers at follow-up and 4 to the group of participants with available sMRI markers at follow-up; and 2 for the semantic memory test (i.e., SRB:1), belonging to the group of participants without available sMRI markers at follow-up.

**Figure 1:**
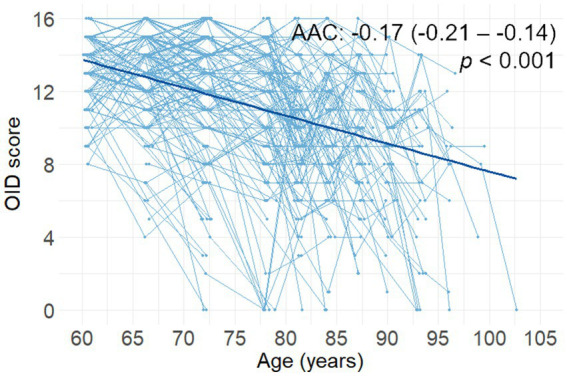
Spaghetti plot (including the regression line) of changes in OID score as a function of age (*n* = 338). Reported β-coefficients (i.e., AAC), 95% CIs, and *p* values were extracted from a linear mixed-effects model including follow-up time from baseline (in years) as a predictor. AAC, annual average change; OID, odor identification.

The OID score decreased steadily during the follow-up period (*p* < 0.001, Figure 1). During the 15-year follow-up period, the average follow-up time for olfactory assessment was 10.4 years (*SD* = 3.6). The mean number of olfactory assessments per person was 2.9 (*SD* = 1.2). The AAC in the OID score was −0.17 items (95% CI, −0.21 to −0.14; *p* < 0.001), in a model controlling for sex, age, and education.

### 3.1 Association between baseline sMRI markers and odor identification decline

Higher global PVS count, lower hippocampal volume, and lower total GM volume at baseline were associated with faster OID decline over the 15-year follow-up period (*p_FWE_* < 0.05, Table 2, *model 1*). When analyzing all MRI markers simultaneously, only higher global PVS count was independently associated with faster OID decline (*p* = 0.031, Table 2, *model 2*), also after additionally controlling for smoking status and semantic memory (*p* = 0.031; Table 2, *model 3*).

**Table 2:** Associations of baseline sMRI markers with annual average change in odor identification score.

	*Model 1*			*Model 2*		*Model 3*	
Baseline sMRI markers^a^	*β*-coefficient (95% CI), AAC in OID score	*p*	*p*_FWE_	*β*-coefficient (95% CI), AAC in OID score	*p*	*β*-coefficient (95% CI), AAC in OID score	*p*
Lacunes presence	−0.082 (−0.177 to 0.012)	0.088	0.196	−0.024 (−0.128 to 0.080)	0.652	−0.024 (−0.128 to 0.080)	0.650
WMH volume	−0.036 (−0.074 to 0.002)	0.063	0.196	0.004 (−0.041 to 0.049)	0.851	0.004 (−0.041 to 0.049)	0.850
Global PVS count	**−0.046 (−0.080 to −0.012)**	**0.008**	**0.040**	**−0.040 (−0.075 to −0.004)**	**0.031**	**−0.039 (−0.075 to −0.004)**	**0.031**
Lateral ventricular volume	−0.039 (−0.078 to −0.000)	0.049	0.196	−0.019 (−0.063 to 0.025)	0.407	−0.019 (−0.063 to 0.025)	0.403
Hippocampal volume	**0.056 (0.018 to 0.093)**	**0.003**	**0.018**	0.038 (−0.014 to 0.089)	0.152	0.038 (−0.014 to 0.089)	0.156
Amygdalar volume	0.024 (−0.014 to 0.061)	0.213	0.213	−0.015 (−0.060 to 0.030)	0.514	−0.015 (−0.060 to 0.030)	0.514
Total GM volume	**0.056 (0.020 to 0.093)**	**0.002**	**0.014**	0.030 (−0.016 to 0.076)	0.199	0.030 (−0.016 to 0.075)	0.203

AAC, annual average change; CI, confidence interval; GM, gray matter; sMRI, structural magnetic resonance imaging; OID, odor identification; PVS, perivascular spaces; WMH, white matter hyperintensities.

*Model 1*, adjusted for demographic factors (sex, age, and education).

*Model 2, all baseline sMRI markers were added simultaneously to model 1.*

*Model 3*, including all baseline sMRI markers simultaneously and adjusted for extra covariates (smoking status and semantic memory performance).

a All sMRI markers were z-transformed except lacunes (presence or absence). Significant results in bold.

There was a significant three-way interaction between global PVS count and follow-up time with age groups. Specifically, higher global PVS count was associated with accelerated OID decline in old-old (≥78 years) but not in young-old participants (<78 years) (*model 1*, *p* for interaction = 0.046, *β*-coefficient, 95% CI, *p*: old-old, −0.098, −0.173 to −0.023, 0.010; young-old, −0.020, −0.058 to 0.018, 0.296). There were significant three-way interactions between hippocampal volume at baseline and follow-up time with sex and *APOE* ε4 status. Lower hippocampal volume was independently associated with accelerated OID decline in males but not in females (*model 2*, *p* for interaction = 0.036, *β*-coefficient, 95% CI, *p*: males, 0.090, 0.014 to 0.166, 0.020; females, −0.022, −0.091 to 0.047, 0.535; *model 3*, *p* for interaction = 0.034, *β*-coefficient, 95% CI, *p*: males, 0.090, 0.014 to 0.166, 0.010; females, −0.023, −0.093 to 0.047, 0.514), and in *APOE* ε4 carriers but not in non-ε4 carriers (*model 2*, *p* for interaction = 0.029, *β*-coefficient, 95% CI, *p*: carriers, 0.138, 0.032 to 0.245, 0.011; non-carriers, 0.005, −0.055 to 0.064, 0.881; *model 3*, *p* for interaction = 0.025, *β*-coefficient, 95% CI, *p*: carriers, 0.143, 0.034 to 0.251, 0.010; non-carriers, 0.004, −0.056 to 0.064, 0.894).

Supplementary analyses showed that a higher deep PVS count, specifically in basal ganglia, was associated with faster OID decline, also after controlling for reduced sleep (*p* < 0.05, Supplementary Tables 2, 3).

### 3.2 Association between sMRI markers progression and odor identification

**Figure 2:**
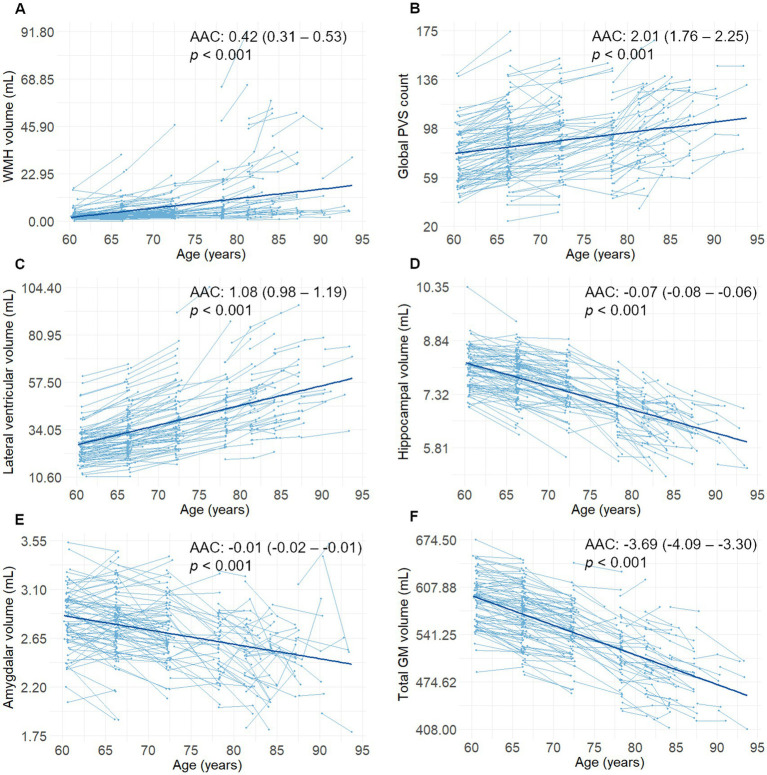
**(A–F)** Spaghetti plots (including the regression lines) of changes in sMRI markers as a function of age (*n* = 225). Reported β-coefficients (i.e., AAC), 95% CIs, and *p* values were extracted from linear mixed-effects models including follow-up time from baseline (in years) as a predictor. AAC, annual average change; GM, gray matter; sMRI, structural magnetic resonance imaging; PVS, perivascular spaces; WMH, white matter hyperintensities.

During the 6-year follow-up period, the average follow-up time for MRI examination was 5.4 years (*SD* = 0.9). The mean number of MRI examinations per person was 2.1 (*SD* = 0.3). Global PVS count, WMH, and lateral ventricular volumes increased steadily during the follow-up period, while hippocampal, amygdalar, and GM volumes decreased steadily during the follow-up period (*p* < 0.001, Figure 2). Lacunes were present in 44 participants (19.6%), of which nine did not present any at baseline. The AAC for WMH volume was 0.42 mL (95% CI, 0.31 to 0.53; *p* < 0.001), for global PVS count was 2.01 (95% CI, 1.76 to 2.25; *p* < 0.001), for lateral ventricular volume was 1.08 mL (95% CI, 0.98 to 1.19; *p* < 0.001), for hippocampal volume was −0.07 mL (95% CI, −0.08 to −0.06; *p* < 0.001), for amygdalar volume was −0.01 mL (95% CI, −0.02 to −0.01; *p* < 0.001), and for total GM volume was −3.69 mL (95% CI, −4.09 to −3.30; *p* < 0.001) in a model controlling for sex, age and education.

Presence of lacunes at follow-up, as well as faster increase of WMH volume and PVS count, lateral ventricular enlargement, and decrease of hippocampal, amygdalar, and total GM volumes during the 6-year follow-up period, were associated with accelerated OID decline over the 15-year follow-up period (*p*_FWE_ < 0.05, Table 3, *model 1*). When entering all sMRI markers into the same model, faster global PVS increase and total GM volume decrease were independently associated with accelerated OID decline (*p* < 0.05, Table 3, *model 2*). The results were equivalent after controlling for smoking status and semantic memory (Table 3, *model 3*).

**Table 3:** Associations of progression in sMRI markers with annual average change in odor identification score.

	*Model 1*			*Model 2*		*Model 3*	
sMRI markers progression^a^	*β*-coefficient (95% CI), AAC in OID score	*p*	*p*_FWE_	*β*-coefficient (95% CI), AAC in OID score	*p*	*β*-coefficient (95% CI), AAC in OID score	*p*
Lacunes presence at follow-up	**−0.147 (−0.256 to −0.037)**	**0.009**	**0.018**	−0.090 (−0.194 to 0.015)	0.093	−0.090 (−0.194 to 0.015)	0.092
WMH volume AAC	**−1.520 (−2.472 to −0.568)**	**0.002**	**0.010**	−0.407 (−1.426 to 0.613)	0.434	−0.417 (−1.437 to 0.603)	0.423
Global PVS count AAC	**−2.105 (−3.813 to −0.397)**	**0.016**	**0.018**	**−1.843 (−3.462 to −0.224)**	**0.026**	**−1.831 (−3.450 to −0.212)**	**0.027**
Lateral ventricular volume AAC	**−1.628 (−2.688 to −0.567)**	**0.003**	**0.010**	−0.578 (−1.747 to 0.591)	0.332	−0.585 (−1.754 to 0.584)	0.327
Hippocampal volume AAC	**1.814 (0.974 to 2.653)**	**<0.001**	**<0.001**	0.916 (−0.041 to 1.872)	0.061	0.923 (−0.033 to 1.880)	0.059
Amygdalar volume AAC	**2.886 (1.077 to 4.695)**	**0.002**	**0.010**	1.095 (−0.801 to 2.991)	0.258	1.073 (−0.824 to 2.970)	0.268
Total GM volume AAC	**2.112 (0.912 to 3.311)**	**0.001**	**0.006**	**1.304 (0.123 to 2.486)**	**0.031**	**1.297 (0.115 to 2.479)**	**0.032**

AAC annual average change, CI confidence interval, GM gray matter, sMRI structural magnetic resonance imaging, OID odor identification, PVS perivascular spaces, WMH white matter hyperintensities.

*Model 1*, adjusted for demographic factors (sex, age, and education) and baseline sMRI markers.

*Model 2, all sMRI markers were added simultaneously to model 1.*

*Model 3*, including all baseline sMRI markers simultaneously and adjusted for extra covariates (smoking status and semantic memory performance).

a AACs were computed using z-transformed sMRI markers. Significant results in bold.

There was a significant three-way interaction between total GM volume and follow-up time with *APOE* ε4 status. Faster total GM decrease was independently associated with accelerated OID decline in *APOE* ε4 carriers but not in non-ε4 carriers (*model 1*, *p* for interaction = 0.003, *β*-coefficient, 95% CI, *p*: carriers, 4.645, 2.596 to 6.695, <0.001, non-carriers, 0.879, −0.574 to 2.331, 0.236; *model 2*, *p* for interaction = 0.001, *β*-coefficient, 95% CI, *p*: carriers, 4.033, 2.056 to 6.010, <0.001, non-carriers, −0.129, −1.565 to 1.306, 0.860). The interaction remained significant after controlling for smoking status and semantic memory (*model 3*, *p* for interaction = 0.001, *β*-coefficient, 95% CI, *p*: carriers, 4.027, 2.054 to 5.999, <0.001, non-carriers, −0. 121, −1.559 to 1.317, 0.869).

Supplementary analyses revealed that a faster accumulation of lobar PVS, specifically in the frontal lobe, was associated with accelerated OID decline, also after controlling for reduced sleep (*p* < 0.05, Supplementary Tables 4, 5).

### 3.3 Sensitivity analyses

When comparing participants with anosmia at baseline (*n* = 13) with the rest of the sample, anosmic participants were older at baseline and showed higher lateral ventricular volume, as well as lower hippocampal, amygdalar, and total GM volumes (*p* < 0.05, Supplementary Tables 6, 7).

After excluding anosmic participants, lower hippocampal and total GM volumes at baseline were associated with faster OID decline (*p_FWE_* < 0.05, Supplementary Table 8, *model 1*). Remarkably, the global PVS count at baseline was no longer associated with faster OID decline after FWE correction (*p* = 0.014, *p_FWE_* > 0.05, Supplementary Table 8, *model 1*). Presence of lacunes at follow-up, faster WMH volume and global PVS increases, ventricular volume enlargement, hippocampal, amygdalar, and total GM volume decreases during the 6-year follow-up, were associated with accelerated OID decline over the 15-year follow-up period (*p_FWE_* < 0.05, Supplementary Table 8, *model 1*). When entering all sMRI markers into the same model, and adjusting for extra covariates, faster global PVS increase and total GM volume decrease were associated with accelerated OID decline (*p* < 0.05, Supplementary Table 8, *models 2* and *3*).

When comparing participants diagnosed with dementia or PD during the 15-year follow-up (*n* = 58) with the rest of the sample, those who received a diagnosis were older at baseline, had fewer years of formal schooling, were more likely to be hypertensive, *APOE* ε4 carriers, and have lacunes, and showed lower performance in global cognition, OID, and semantic memory, larger WMH and lateral ventricular volumes, as well as smaller hippocampal, amygdalar, and total GM volumes (*p* < 0.05, Supplementary Table 9). Moreover, they displayed an accelerated OID decline, WMH volume increase, lateral ventricular volume enlargement, as well as hippocampal and amygdalar volume decreases (*p* < 0.05, Supplementary Table 7).

After excluding participants diagnosed with dementia or PD during follow-up, there were no statistically significant associations between baseline sMRI markers and OID decline (*p* > 0.05, Supplementary Table 10). Faster increase of WMH volume, lateral ventricular volume, and faster decrease of hippocampal and total GM volumes during the 6-year follow-up, were associated with accelerated OID decline over the 15-year follow-up period (*p_FWE_* < 0.05, Supplementary Table 10, *model 1*). Faster increase in global PVS count was associated with accelerated OID decline after introducing all markers into the same model and controlling for smoking status and semantic memory (*p* < 0.05, Supplementary Table 10, *models 2* and *3*).

## 4 Discussion

In this population-based cohort study, we investigated associations of sMRI markers suggesting microvascular lesions and neurodegeneration with OID decline across 15 years. We found that (1) higher PVS count and lower hippocampal and total GM volumes at baseline, and (2) occurrence of lacunes over follow-up, faster increase of WMH volume, global PVS count, and lateral ventricular enlargement, hippocampal, amygdalar, and total GM atrophy were associated with accelerated OID decline. While previous studies mainly pointed to the involvement of brain atrophy process in age-related olfactory decline, our results added to the current literature by suggesting the contribution of cerebral microvascular lesions to an accelerated OID decline.

Our findings on the relationship between markers of cerebral microvascular lesions and OID decline provide new insights into the potential mechanisms underlying olfactory deficits. We found that a higher PVS count at baseline and its increase over time are associated with an accelerated OID decline. PVS are fluid-filled spaces surrounding cerebral small vessels, which are involved in the clearance of soluble waste products (Gouveia-Freitas and Bastos-Leite, 2021). It has been proposed that a reduced outflow through arterioles due to microvascular changes (e.g., atherosclerosis, arteriolosclerosis, elastin dysfunction) may alter their functioning, resulting in the accumulation of waste products and their enlargement (Gouveia-Freitas and Bastos-Leite, 2021). Recent neuropathological findings suggest that visible (i.e., enlarged) PVS may reflect an impaired clearance of beta-amyloid protein, contributing to its accumulation in the underlying cerebral cortex and promoting cerebral amyloid angiopathy (Perosa et al., 2022). Thus, the observed associations of OID decline with PVS and their increased count over time may reflect both microvascular burden and AD-related protein deposition in key olfactory areas in aging. In addition, it is suggested that PVS enlargement might occur before the accumulation of other microvascular lesions and neurodegeneration (Francis et al., 2019; Li et al., 2022), and it has emerged as an early predictor of progression to dementia (Paradise et al., 2021; Romero et al., 2022). Considering this perspective, the decline in olfactory function might hold significance as an early sign of structural brain aging.

To go further, we explored the regional counts of PVS and found that their count in basal ganglia and their accumulation in the frontal lobe are associated with accelerated OID decline. Visible PVS in the basal ganglia were linked previously to cerebral ischemia (Gouveia-Freitas and Bastos-Leite, 2021), which aligns with the hypothesized cerebrovascular mechanism of hypoperfusion causing vascular damage due to atherosclerosis of the supplying blood vessels (Schubert et al., 2015; Siegel et al., 2019). On the other hand, brain atrophy might also contribute to PVS enlargement by ex-vacuo dilatation secondary to shrinkage of cerebral tissue, even though there is mixed evidence and the underlying mechanism remains controversial (Zhang et al., 2016). In our study, the relationships between enlarged PVS and OID decline might be considered independent of brain atrophy since the results for baseline counts and progression remain significant after introducing all markers in the same model. Notably, in a sample of individuals with PD from the Parkinson’s Progression Marker Initiative (PPMI) cohort, a higher PVS count in the basal ganglia was associated with worse OID (Fang et al., 2020). Indeed, beyond the specific underlying processes, a presumable basal ganglia dysfunction associated with OID and its decline is coherent with evidence of their involvement in higher-order olfactory functions (i.e., odor encoding and recognition) (Eek et al., 2023). Similar mechanisms may be behind the association of OID with the accumulation of PVS in the frontal lobe, which encompasses some key olfactory regions (e.g., orbitofrontal and piriform cortices) structurally and functionally related to poorer olfactory functions in aging (Felix et al., 2020; Pellegrino et al., 2016; Shen et al., 2013; Seubert et al., 2020; Wu et al., 2019). Following this approach, future research should address whether different types of lesions beyond PVS may contribute to olfactory decline depending on the affected structures.

We also investigated if sleep quality at baseline could impact the relation between PVS and OID decline. After adjusting the analyses for self-reported reduced sleep, the results remained the same. Future studies should address the impact of objective sleep parameters, beyond the use of self-reported sleep measures, on the dysfunction of the glymphatic system, as a potential mechanism for olfactory deterioration in aging, as recently reported regarding memory decline (Ma et al., 2024).

In addition, the presence of lacunes at follow-up and the accelerated increase of WMH volume were associated with faster OID decline, aligning with the hypothesis of olfactory deficits as a consequence of vascular damage in key olfactory processing areas (Schubert et al., 2015; Siegel et al., 2019). This hypothesis is supported by evidence of associations of carotid intima-media thickness and atherosclerosis with OID decline in humans and middle cerebral artery occlusion causing olfactory cortex infarcts in rats (Duverger and MacKenzie, 1988; Schubert et al., 2015). In contrast, it is noteworthy that previous population-based studies have shown mixed results when analyzing possible cross-sectional relations between WMH and olfactory function (Devanand et al., 2010; Buchanan et al., 2020; Dong et al., 2023). In this regard, our results revealed no link between baseline WMH volume and OID decline, which aligns with those studies using cross-sectional designs that found no association (Devanand et al., 2010; Buchanan et al., 2020). The absence of a relationship between baseline WMH burden and OID decline contrasts with the observed longitudinal relationship. The divergence of results may be influenced by the large variability of WMH, OID, and their trajectories in aging, where, an acceleration may signal a more severe pathological process, such as an impending dementia disorder (Silbert et al., 2012; Guo et al., 2023; Pacyna et al., 2023).

Regarding neurodegeneration markers, we found an association between lower hippocampal volume at baseline and faster OID decline, complementing prior findings showing that OID is positively related to hippocampal volume and integrity in aging (Seubert et al., 2020; Shrestha et al., 2023; Diez et al., 2024), and that OID dysfunction is associated with lower hippocampal volume (Dintica et al., 2019) and faster hippocampal atrophy (Tian et al., 2023). Furthermore, lower total GM volume at baseline was associated with accelerated OID decline, which aligns with a widespread pattern of reduced GM volume found in faster decliners, including olfactory and other AD-related regions (Pacyna et al., 2023). This finding is also congruent with associations between GM measures in other regions beyond MTL and poorer OID in CN older individuals (Bothwell et al., 2023; Diez et al., 2024; Shrestha et al., 2023; Tian et al., 2023).

Another contribution of our study is that we investigated the relation between longitudinal brain atrophy trajectories and future OID decline. We found a relationship between faster hippocampal, amygdalar, and global atrophy and accelerated OID decline. Overall, this finding reveals that an early atrophic process, affecting specifically AD-related areas, is associated with olfactory deterioration in aging, as prior cross-sectional evidence suggested (Devanand et al., 2010; Dintica et al., 2019; Seubert et al., 2020; Pacyna et al., 2023; Shrestha et al., 2023; Tian et al., 2023). This result complements the 5-year associations of volume decreases in specific hippocampal subfields with OID decline previously reported (Menelaou et al., 2022). Relatedly, a recent study reported that poorer OID was associated cross-sectionally with lower GM matter volume and longitudinally with tau accumulation in key olfactory areas, including MTL structures in CN older adults (Diez et al., 2024). In addition, the authors found that tau spreading in the brain followed a pathway from MTL structures toward the olfactory system (Diez et al., 2024). Following this direction, future studies should investigate which early regional brain atrophy trajectories underlie OID decline. One promising path to address this question is exploring the longitudinal trajectories of core regions of the functional olfactory network, following previous cross-sectional studies exploring these regions in relation to OID and episodic memory performance (Seubert et al., 2020; Jobin et al., 2023a).

Interestingly, some associations did not remain significant when entering all sMRI markers in the same model. This finding may reflect a joint effect of cerebral microvascular lesions and neurodegeneration on olfactory decline. Especially, considering their interactive effects (Paolini Paoletti et al., 2021) and that most dementia cases in community-based cohorts and brain banks exhibit mixed dementia pathology (Schneider et al., 2007; Agrawal and Schneider, 2022; Robinson et al., 2023). The interpretation should be cautious, especially for sMRI markers suggesting microvascular lesions. For example, neuropathological evidence showed that WMH corresponds to areas of demyelination and gliosis (i.e., neuronal loss) (Young et al., 2008), and a recent study indicated that WMH contributes to ventricular enlargement (Jochems et al., 2022). Regarding enlarged PVS, as we highlighted above, their formation may be due to an initial composition alteration of the perivascular fluid (Gouveia-Freitas and Bastos-Leite, 2021) or be related to brain atrophy (Zhang et al., 2016). Future studies should address how these different processes could interact in their association with olfactory deterioration in aging and predementia stages through implementing mechanist models.

We detected a significant three-way interaction between sMRI markers, follow-up time, and age groups. Higher baseline PVS count was more strongly associated with accelerated OID decline in old-old adults, who have previously demonstrated a faster decline (Ekström et al., 2020). This interaction with baseline PVS may reflect the broader relationship between PVS accumulation and olfactory decline, suggesting that the observed age-specific effect is a more pronounced manifestation of this overall pattern. On the other hand, we found that lower hippocampal volume at baseline was more strongly associated with accelerated OID decline in males. It is common knowledge that sex impacts olfactory function, with females outperforming males (Sorokowski et al., 2019). Moreover, sex differences in brain olfactory regions have also been reported (Alotaibi et al., 2023), including lower GM concentration in the hippocampi in males (Garcia-Falgueras et al., 2006). Despite this, longitudinal analyses point out that hippocampal atrophy appears to impact olfactory deterioration in both sexes. Finally, lower hippocampal volume at baseline and faster GM atrophy in *APOE* ε4 carriers were associated with accelerated OID decline, which may reflect an ongoing pathological process and early functional changes in a group that already presents a high AD genetic risk (Calhoun-Haney and Murphy, 2005; Mishra et al., 2018). Indeed, *APOE* ε4 has been reported to be associated with smaller volumes of specific olfactory areas (i.e., amygdalae and entorhinal cortex) (Pacyna et al., 2023).

It is noteworthy that the main findings remained significant after controlling for current smoking and semantic memory performance. These results point to central nervous system mechanisms independent of smoking effects on the olfactory function (Ajmani et al., 2017). On the other hand, they reinforce the notion that memory has a significant but modest effect on OID (Jobin et al., 2023a), despite potentially shared mechanisms underlying their decline, such as MTL atrophy (Bettio et al., 2017; Bothwell et al., 2023).

In the sensitivity analyses excluding anosmic individuals, the global PVS count at baseline was no longer associated with OID decline, while the PVS increase remained significant. It is important to note that participants classified as anosmic still display olfactory decline, which did not significantly differ from the observed in those without anosmia. We further found that the associations of baseline brain sMRI markers with OID decline did not remain significant after excluding individuals diagnosed with dementia or PD during follow-up. Of note, those participants with incident syndromes had lower OID and cognitive scores and displayed a worse profile for microvascular lesions and neurodegeneration at baseline. Moreover, they showed a faster decline in their ability to correctly identify smells. Thus, these individuals were likely in an advanced early stage of neurodegeneration, characterized by ongoing structural and functional pathological processes, which might explain why removing them from the sample led to nonsignificant associations with the baseline sMRI markers. In contrast, the longitudinal associations for all markers remained significant after the exclusion of these participants, except for the presence of lacunes and amygdalar atrophy.

Previous research has mainly targeted olfactory performance as a marker of future brain atrophy and dementia incidence (Adams et al., 2018; Laukka et al., 2023; Pacyna et al., 2023; Tian et al., 2023). The current findings suggest that olfactory dysfunction potentially develops in parallel with changes related to both cerebrovascular burden and neurodegeneration, which occur early in aging and dementia development, some of them affecting key areas for high-order olfactory and cognitive functions (i.e., hippocampi, basal ganglia, and frontal lobe). In this regard, we collected new evidence supporting central nervous system changes as a potential mechanism of OID decline in aging (i.e., hippocampal atrophy) and propose new candidates (i.e., PVS enlargement in basal ganglia and frontal lobe) for which we may expect an overacceleration in the progression to dementia. Future studies should address the risk of developing olfactory dysfunction in aging based on MRI biomarkers, as it predicts future dementia (Adams et al., 2018; Murphy, 2019; Laukka et al., 2023).

The major strength of this study is the population-based design with a long follow-up period for brain sMRI markers (6 years) and OID (15 years), which allowed us to analyze their longitudinal associations. Significantly, we extended previous studies focusing on neurodegeneration by adding cerebrovascular burden markers. A possible limitation is the use of diverse automated and manual approaches for the assessment of sMRI markers, which could affect the replicability in different samples. Furthermore, the regional component was not extensively addressed for other markers apart from subcortical MTL regions and PVS.

The present study lacks protein biomarkers of AD pathology. Of interest, a population-based study in CN older adults linked faster accumulation of beta-amyloid in the orbitofrontal cortex and tau in the entorhinal cortex to accelerated OID decline over 2.4 years (Tian et al., 2022). Future studies should longitudinally explore the link between olfactory function, microvascular lesions, and neurodegeneration also including a range of available molecular markers. Considering multiple pathological processes may result in models bringing us a more detailed picture of which brain changes can lead to olfactory decline while outlining high-probability trajectories of conversion to dementia. The resulting models could be transferable to clinical settings through early detection approaches by combining olfactory assessment with, for example, blood-based markers (Xiao et al., 2023). Another constraint is the participants’ profile, which encompasses an older population residing in the central area of Stockholm, relativity healthy and fit, predominantly high socioeconomic standing, and Swedish-born. These characteristics might limit the generalizability of our findings.

## 5 Conclusion

Overall, in this population-based cohort study, we report a longitudinal association of the progression of microvascular lesions and neurodegeneration with OID deterioration in aging. Further research should model how the longitudinal interplay of different brain pathological processes may impact the sense of smell. These mechanistic models may increase our understanding of olfactory decline in aging and the period leading up to a dementia diagnosis, facilitating early dementia detection.

## Data Availability

The data analyzed in this study is subject to the following licenses/restrictions: Data related to the current study were derived from SNAC-K. Access to these anonymized data are available upon reasonable request and approval by the SNAC-K data management and maintenance committee at the Aging Research Center, Karolinska Institutet, Stockholm, Sweden. Requests to access these datasets should be directed to https://www.snac-k.se/for-researchers/application-form/.
