# Corrigendum: Cerebrovascular burden and neurodegeneration linked to 15-year odor identification decline in older adults

**DOI:** 10.3389/fnagi.2025.1599273

**Published:** 2025-04-16

**Authors:** Javier Oltra, Grégoria Kalpouzos, Ingrid Ekström, Maria Larsson, Yuanjing Li, Chengxuan Qiu, Erika J. Laukka

**Affiliations:** ^1^Aging Research Center, Department of Neurobiology, Care Sciences and Society, Karolinska Institutet and Stockholm University, Stockholm, Sweden; ^2^Gösta Ekman Laboratory, Department of Psychology, Stockholm University, Stockholm, Sweden; ^3^Stockholm Gerontology Research Center, Stockholm, Sweden

**Keywords:** olfaction, microvascular lesions, brain atrophy, population-based study, aging, dementia

In the published article, there was an error in the affiliation of Erika J. Laukka. Instead of affiliation 2 and 3, (^2^Gösta Ekman Laboratory, Department of Psychology, Stockholm University, Stockholm, Sweden; ^3^Stockholm Gerontology Research Center, Stockholm, Sweden), Erika J. Laukka should have instead been affiliated with 1 and 3 (^1^Aging Research Center, Department of Neurobiology, Care Sciences and Society, Karolinska Institutet and Stockholm University, Stockholm, Sweden; ^3^Stockholm Gerontology Research Center, Stockholm, Sweden).

The authors apologize for this error and state that this does not change the scientific conclusions of the article in any way. The original article has been updated.

