# Supplementary material for: Cerebrovascular burden and neurodegeneration linked to 15-year odor identification decline in older adults
Source: Front Aging Neurosci. 2025 Mar 24;17:1539508. doi: 10.3389/fnagi.2025.1539508 (PMC11973317; doi:10.3389/fnagi.2025.1539508)
Supplement: Supplementary file 1 [file Table_1.docx]

Supplementary Material

**Supplementary Methods**

**Neuropsychological assessment**

*Semantic memory*. Vocabulary was assessed using the 30-item multiple-choice Swedish vocabulary test from the Synonym Reasoning Blocks (Dureman, 1960; Nilsson et al., 1997). General knowledge was assessed using 10 general knowledge questions (Dahl et al., 2009). Scores were computed as the number of correct responses.

*Episodic memory*. The episodic memory task was tailored for SNAC-K and consisted of a list of 16 unrelated concrete nouns. The word free recall and recognition scores were computed as the number of correctly recalled and recognized words (Laukka et al., 2013).

*Attention and executive function*. The attention and executive function tasks were the Trail Making Test-A and -B. The TMT-A and TMT-B scores were computed as the completion times in seconds (Tombaugh, 2004).

*Language*. Language tasks consisted of two letter fluency (F and A) and two category fluency (animals and professions) 1-minute tasks (Tombaugh et al., 1999). For letter and category fluency, scores consisted of the average number of words generated.

*Visuospatial abilities*. The visuospatial abilities task consisted of a 10-item version of the Shepherd–Metzler mental rotation test (Vandenberg and Kuse, 1978; Rehnman and Herlitz, 2006). The score was computed as the number of correct responses.

*Perceptual speed*. Pattern comparison consisted of judging if 30 different pairs (distributed into two trials of 15) of line-segment patterns were identical or different (Salthouse and Babcock, 1991). The score was the mean number of correct classifications within 30 seconds for the two trials. Digit cancellation consisted of inspecting 11 rows of random digits (range 1 to 9) and crossing every digit 4 detected (target) (Zazzo, 1974). The score was the number of digits correctly crossed within 30 seconds.

**References**

Dahl, M., Allwood, C. M., and Hagberg, B. (2009). The realism in older people’s confidence judgments of answers to general knowledge questions. Psychol Aging 24, 234–238. doi: 10.1037/a0014048

Dureman, I. (1960). SRB: 1. Stockholm: Psykologiförlaget.

Laukka, E. J., Lövdén, M., Herlitz, A., Karlsson, S., Ferencz, B., Pantzar, A., et al. (2013). Genetic effects on old-age cognitive functioning: a population-based study. Psychol Aging 28, 262–274. doi: 10.1037/a0030829

Nilsson, L.-G., Bäckman, L., Erngrund, K., Nyberg, L., Adolfsson, R., Bucht, G., et al. (1997). The betula prospective cohort study: Memory, health, and aging. Aging Neuropsychol and Cogn 4, 1–32. doi: 10.1080/13825589708256633

Rehnman, J., and Herlitz, A. (2006). Higher face recognition ability in girls: Magnified by own-sex and own-ethnicity bias. Memory 14, 289–296. doi: 10.1080/09658210500233581

Salthouse, T. A., and Babcock, R. L. (1991). Decomposing adult age differences in working memory. Dev Psychol 27, 763–776. doi: 10.1037/0012-1649.27.5.763

Tombaugh, T. N. (2004). Trail Making Test A and B: normative data stratified by age and education. Arch Clin Neuropsychol 19, 203–214. doi: 10.1016/S0887-6177(03)00039-8

Tombaugh, T. N., Kozak, J., and Rees, L. (1999). Normative data stratified by age and education for two measures of verbal fluency: FAS and animal naming. Arch Clin Neuropsychol 14, 167–177.

Vandenberg, S. G., and Kuse, A. R. (1978). Mental rotations, a group test of three-dimensional spatial visualization. Percept Mot Skills 47, 599–604. doi: 10.2466/pms.1978.47.2.599

Zazzo, R. (1974). Test Des Deux Barrages. Actualités Pedagogiques Et Psychologiques, Vol. 7. Neuchâtel: Delachaux et Niestlé.

**
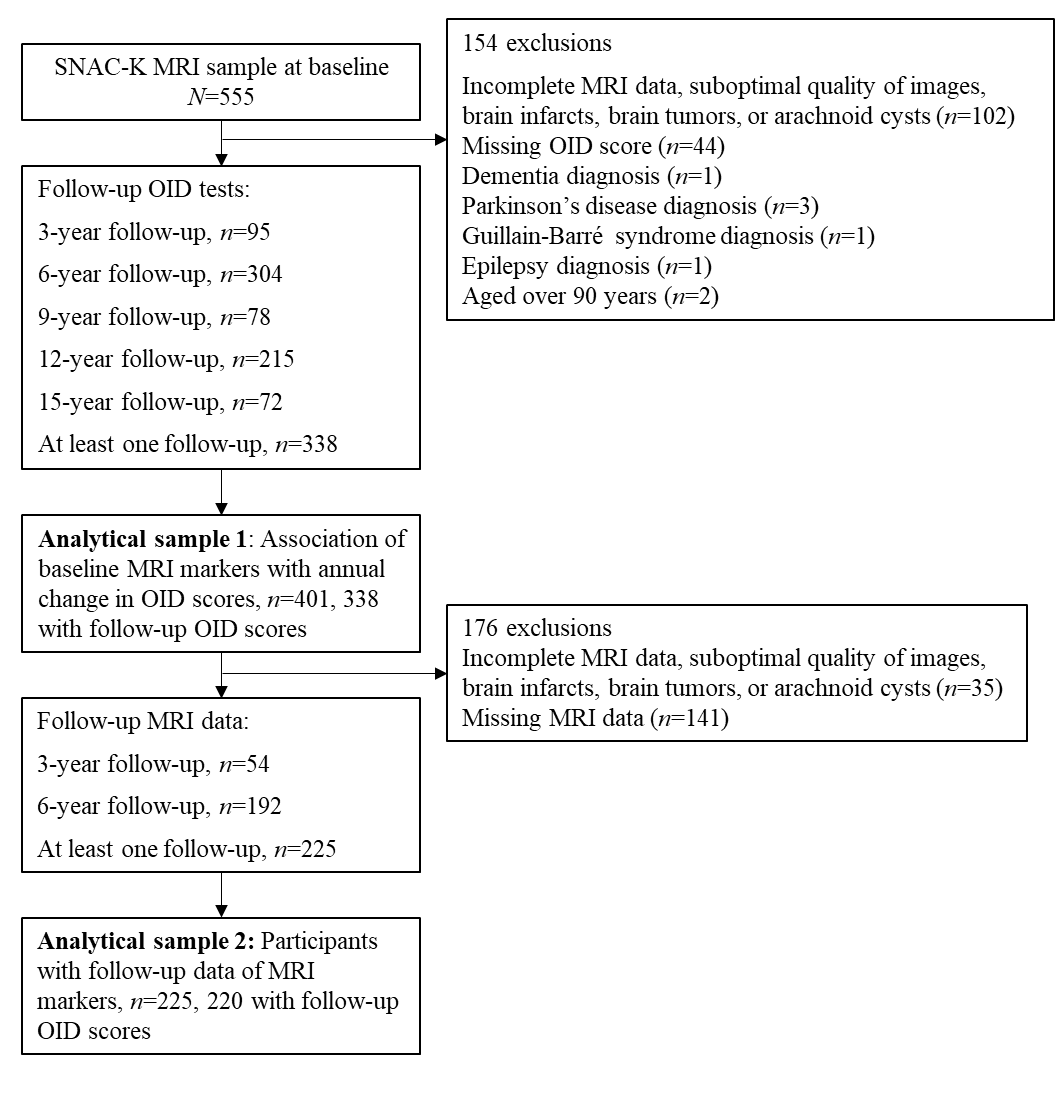
**

**Supplementary Figure 1** Flow chart of study participation. *SNAC-K* Swedish National Study on Aging and Care in Kungsholmen, *OID* odor identification, *MRI* magnetic resonance imaging.

|  | | | Total sample | Follow-up sMRI markers | | |
| --- | --- | --- | --- | --- | --- | --- |
| Cognitive tests | | | (*n*=401) | No (*n*=176) | Yes (*n*=225) | *p* |
| Semantic memory | | |  |  |  |  |
|  | Vocabulary^†^ | | 23.6 (4.3) | 23.1 (4.9) | 24.0 (3.8) | 0.040 |
|  | General knowledge^†^ | | 7.1 (1.5) | 6.9 (1.6) | 7.2 (1.5) | 0.129 |
| Episodic memory | | |  |  |  |  |
|  | Word free recall | | 7.4 (2.2) | 7.2 (2.4) | 7.5 (2.1) | 0.026 |
|  | Word recognition | | 11.6 (2.8) | 11.2 (3.0) | 11.9 (2.6) | 0.198 |
| Attention and executive function | | |  |  |  |  |
|  | TMT-A, time (s)^†^ | | 13.9 (5.7) | 14.9 (7.1) | 13.0 (4.1) | <0.001 |
|  | TMT-B, time (s)^†^ | | 27.3 (12.4) | 29.9 (13.3) | 25.4 (11.3) | <0.001 |
| Language | | |  |  |  |  |
|  | | Letter fluency^†^ | 14.7 (4.5) | 14.3 (4.5) | 15.0 (4.5) | 0.130 |
|  | | Category fluency^†^ | 19.9 (4.8) | 19.3 (4.8) | 20.3 (4.8) | 0.035 |
| Visuospatial abilities | | |  |  |  |  |
|  | | Mental rotations^†^ | 6.3 (2.0) | 6.2 (1.9) | 6.4 (2.0) | 0.280 |
| Perceptual speed | | |  |  |  |  |
|  | | Pattern comparison^†^ | 14.9 (3.5) | 14.2 (3.8) | 15.5 (3.1) | <0.001 |
|  | | Digit cancellation^†^ | 18.6 (4.1) | 18.2 (4.0) | 18.9 (4.2) | 0.034 |

**Supplementary Table 1** Cognitive performance of study participants in the total sample and by availability of sMRI markers at follow-up

Data are presented as *M* (*SD*). *M* mean, *TMT* Trail Making Test, *SD* standard deviation.

^†^ The number of participants with missing values was 2 for vocabulary (without available follow-up sMRI markers), 2 for general knowledge (1 without available follow-up sMRI markers), 5 for TMT-A (2 without available follow-up sMRI markers), 32 for TMT-B (16 without available follow-up sMRI markers), 2 for letter fluency (1 without available follow-up sMRI markers), 2 for category fluency (without available follow-up sMRI markers), 3 for mental rotations (2 without available follow-up sMRI markers), 3 for pattern comparison (2 without available follow-up sMRI markers), 4 for digit cancellation (3 without available follow-up sMRI markers).

**Supplementary Table 2** Perivascular spaces count at baseline by the analyzed brain regions

| Brain regions | | | PVS count, mean (*SD*) |
| --- | --- | --- | --- |
| Global | | | 82.9 (26.1) |
|  | Lobar area | | 50.0 (21.0) |
|  | Infratentorial area | | 2.6 (1.8) |
|  | Deep area | | 30.3 (8.9) |
|  |  | Basal ganglia | 20.2 (7.0) |
|  |  | Sub-insular region | 7.1 (3.3) |
|  |  | Hippocampus | 2.9 (2.1) |

*PVS* perivascular spaces, *SD* standard deviation

**Supplementary Table 3** Associations of baseline perivascular spaces count with annual average change in odor identification

|  | | *Model 1* |  | *Model 2* |  |
| --- | --- | --- | --- | --- | --- |
| Baseline PVS count | | β-coefficient (95% CI),  AAC in OID score | *p* | β-coefficient (95% CI),  AAC in OID score | *p* |
| *1^st^ set* | |  |  |  |  |
|  | Global | **-0.046 (-0.080 – -0.012)** | **0.008** | **-0.047 (-0.082 – -0.013)** | **0.007** |
| *2^nd^ set* | |  |  |  |  |
|  | Lobar | -0.022 (-0.059 – 0.016) | 0.255 | -0.022 (-0.060 – 0.016) | 0.265 |
|  | Infratentorial | 0.003 (-0.033 – 0.040) | 0.865 | 0.001 (-0.036 – 0.038) | 0.959 |
|  | Deep | **-0.042 (-0.080 – -0.004)** | **0.028** | **-0.042 (-0.080 – -0.004)** | **0.030** |
| *3^rd^ set* | |  |  |  |  |
|  | Basal ganglia | **-0.046 (-0.082** – **-0.010)** | **0.013** | **-0.046 (-0.082** – **-0.009)** | **0.014** |
|  | Sub-insular | -0.008 (-0.043 – 0.027) | 0.662 | -0.009 (-0.045 – 0.027) | 0.626 |
|  | Hippocampal | -0.012 (-0.048 – 0.025) | 0.523 | -0.011 (-0.048 – 0.026) | 0.558 |

*CI* confidence interval, *OID* odor identification, *PVS* perivascular spaces.

*1^st^ set* included global PVS count. *2^nd^ set* included lobar, infratentorial and deep PVS counts. *3^rd^ set* included basal ganglia, sub-insular, and hippocampal PVS counts.

*Model 1*, adjusted for demographic factors (sex, age, education).

*Model 2*, adjusted for demographic factors (sex, age, education) and reduced sleep.

Significant results in bold.

**Supplementary Table 4** Annual average change for perivascular spaces count at baseline in the analyzed regions

| Brain regions | | AAC (95% CI) | *p* |
| --- | --- | --- | --- |
| Global | | 2.01 (1.76 – 2.25) | < 0.001 |
| Lobar | | 1.40 (1.20 – 1.59) | < 0.001 |
|  | Frontal | 0.92 (0.78 – 1.05) | < 0.001 |
|  | Parieto-occipital | 0.48 (0.36 – 0.59) | < 0.001 |
| Infratentorial | | 0.03 (0.01 – 0.05) | 0.006 |
| Deep | | 0.58 (0.47 – 0.68) | < 0.001 |

*AAC* annual average change, *PVS* perivascular spaces, *CI* confidence interval.

**Supplementary Table 5** Associations of annual average changes in regional perivascular spaces counts with annual average change in odor identification

|  | | *Model 1* |  | *Model 2* |  |
| --- | --- | --- | --- | --- | --- |
| PVS count AAC^a^ | | β-coefficient (95% CI),  AAC in OID score | *p* | β-coefficient (95% CI),  AAC in OID score | *p* |
| *1^st^ set* | |  |  |  |  |
|  | Global | **-2.105 (-3.813 – -0.397)** | **0.016** | **-2.105 (-3.834 – -0.376)** | **0.017** |
| *2^nd^ set* | |  |  |  |  |
|  | Lobar | **-2.396 (-4.163 – -0.629)** | **0.008** | **-2.423 (-4.206 – -0.641)** | **0.008** |
|  | Infratentorial | -0.212 (-0.968 – 0.543) | 0.582 | -0.204 (-0.965 – 0.556) | 0.599 |
|  | Deep | -0.011 (-1.779 – 1.758) | 0.991 | 0.033 (-1.754 – 1.821) | 0.971 |
| *3^rd^ set* | |  |  |  |  |
|  | Frontal | **-2.150 (-4.061 – -0.240)** | **0.027** | **-1.981 (-3.952 – -0.010)** | **0.049** |
|  | Parieto-occipital | -0.952 (-2.362 **–** 0.458) | 0.186 | -1.118 (-2.572 **–** 0.336) | 0.132 |

*CI* confidence interval, *OID* odor identification, *PVS* perivascular spaces.

*1st set* included global PVS count AAC. *2nd set* included lobar, infratentorial and deep PVS count AACs. *3rd set* included frontal and parieto-occipital PVS count AACs.

Model 1, adjusted for demographic factors (sex, age, education).

Model 2, adjusted for demographic factors (sex, age, education) and reduced sleep.

^a^ AACs were computed using z-transformed PVS counts.

Significant results in bold.

**Supplementary Table 6** Participant characteristics at baseline by presence of anosmia and availability of MRI markers at follow-up

|  | | | Anosmia at baseline | | | |  | |  | | Follow-up sMRI markers in individuals without anosmia at baseline | | | | | |
| --- | --- | --- | --- | --- | --- | --- | --- | --- | --- | --- | --- | --- | --- | --- | --- | --- |
| Characteristics | | | Yes (*n*=13) | | No (*n*=388) | | *p* | |  | | No (*n*=169) | | Yes (*n*=219) | | *p* | |
| Age (y), *M* (*SD*) | | | 80.2 (7.0) | | 69.9 (8.5) | | <0.001 | |  | | 71.0 (9.0) | | 69.9 (8.1) | | 0.016 | |
| Age groups | | |  | |  | |  | |  | |  | |  | |  | |
|  | Young-old (<78), *n* (%) | | | 2 (15.4) | | 280 (72.2) | | <0.001 | |  | | 117 (69.2) | | 163 (74.4) | | 0.257 |
|  | Old-old (≥78), *n* (%) | | 11 (84.6) | | 108 (27.8) | |  | |  | | 52 (30.8) | | 56 (25.6) | |  | |
| Female, *n* (%) | | | 9 (69.2) | | 231 (59.5) | | 0.483 | |  | | 95 (56.2) | | 136 (62.1) | | 0.241 | |
| Education (y), *M* (*SD*) | | | 11.0 (3.7) | | 12.8 (4.3) | | 0.105 | |  | | 12.1 (4.2) | | 13.3 (4.3) | | 0.006 | |
| MMSE score, *M* (*SD*) | | | 28.8 (1.5) | | 29.1 (1.0) | | 0.507 | |  | | 29.0 (1.1) | | 29.2 (1.0) | | 0.083 | |
| Hypertension, *n* (%) | | | 6 (46.2) | | 164 (42.3) | | 0.780 | |  | | 75 (44.4) | | 89 (40.6) | | 0.460 | |
| Diabetes mellitus, *n* (%) | | | 0 (0.0) | | 29 (7.5) | | 0.612 | |  | | 15 (8.9) | | 13 (5.9) | | 0.267 | |
| Current smoking, *n* (%) | | | 1 (7.7) | | 56 (14.4) | | 0.703 | |  | | 28 (16.6) | | 28 (12.8) | | 0.293 | |
| *APOE* ε4 allele | | |  | |  | |  | |  | |  | |  | |  | |
|  | | Non carrier, *n* (%) | 9 (69.2) | | 267 (68.8) | | 1.000 | |  | | 113 (66.9) | | 154 (70.3) | | 0.887 | |
|  | | Carrier, *n* (%) | 4 (30.8) | | 109 (28.1) | |  | |  | | 47 (27.8) | | 62 (28.3) | |  | |
|  | | Missing, *n* (%) | 0 (0.0) | | 12 (3.1) | |  | |  | | 9 (5.3) | | 3 (1.4) | |  | |
| OID score, *M* (*SD*) | | | 4.8 (1.7) | | 12.3 (2.2) | | <0.001 | |  | | 12.2 (2.2) | | 12.4 (2.2) | | 0.319 | |
| Semantic memory score, *M* (*SD*)^†^ | | | 21.7 (4.9) | | 23.7 (4.3) | | 0.103 | |  | | 23.1 (4.9) | | 24.1 (3.8) | | 0.031 | |
| eTIV (mL), *M* (*SD*) | | | 1445.8 (149.9) | | 1495.0 (155.0) | | 0.256 | |  | | 1501.2 (149.1) | | 1490.2 (159.6) | | 0.491 | |
| sMRI markers | | |  | |  | |  | |  | |  | |  | |  | |
|  | | Presence of lacunes, *n* (%) | 5 (38.5) | | 67 (17.3) | | 0.064 | |  | | 32 (18.9) | | 35 (16.0) | | 0.445 | |
|  | | WMH volume (mL), *M* (*SD*) | 6.6 (3.8) | | 7.1 (9.6) | | 0.108 | |  | | 8.2 (10.4) | | 6.2 (8.9) | | 0.047 | |
|  | | Global PVS count, *M* (*SD*) | 77.2 (29.9) | | 83.0 (25.9) | | 0.425 | |  | | 84.7 (28.4) | | 81.8 (23.9) | | 0.281 | |
|  | | Lateral ventricular volume (mL), *M* (*SD*) | 44.7 (9.7) | | 37.9 (16.3) | | 0.025 | |  | | 41.5 (17.9) | | 35.1 (14.5) | | <0.001 | |
|  | | Hippocampal volume (mL), *M* (*SD*) | 7.0 (0.9) | | 7.5 (0.8) | | 0.023 | |  | | 7.4 (0.8) | | 7.6 (0.8) | | 0.007 | |
|  | | Amygdalar volume (mL), *M* (*SD*) | 2.5 (0.4) | | 2.7 (0.3) | | 0.008 | |  | | 2.7 (0.3) | | 2.7 (0.3) | | 0.236 | |
|  | | Total GM volume (mL), *M* (*SD*) | 495.7 (49.0) | | 553.1 (51.4) | | <0.001 | |  | | 547.3 (53.2) | | 557.6 (49.7) | | 0.052 | |

*eTIV* estimated total intracranial volume, *GM* gray matter, *M* mean, *MMSE* Mini Mental State Examination, *OID* odor identification, *PVS* perivascular spaces, *SD* standard deviation, *sMRI* structural magnetic resonance imaging, *WMH* white matter hyperintensities.

^†^ The number of participants with missing values was 2 for the semantic memory test (i.e., SRB:1), belonging to the group of participants no anosmic at baseline without available sMRI markers at follow-up.

|  | Participants with anosmia at baseline | Participants without anosmia at baseline | *p* |
| --- | --- | --- | --- |
| OID AAC | -0.080 (-0.386 – 0.230) | -0.177 (-0.214 – -0.140) | 0.602 |
| Presence of lacunes at follow-up | 2 (28.6) | 42 (19.3) | 0.625 |
| WMH volume AAC | 0.417 (0.083 – 0.751) | 0.420 (0.307 – 0.533) | 0.764 |
| Global PVS count AAC | 2.292 (1.336 – 3.247) | 2.009 (1.759 – 2.261) | 0.748 |
| Lateral ventricular volume AAC | 1.155 (0.690 – 1.619) | 1.083 (0.973 – 1.193) | 0.866 |
| Hippocampal volume AAC | -0.089 (0.123 – -0.055) | -0.072 (-0.081 – -0.063) | 0.718 |
| Amygdalar volume AAC | -0.008 (-0.046 – 0.029) | -0.015 (-0.020 – -0.010) | 0.797 |
| Total GM volume AAC | -2.797 (-4.204 – -1.390) | -3.715 (-4.117 – -3.313) | 0.587 |
|  | Participants diagnosed with dementia or PD at follow-up | Participants without dementia or PD | *p* |
| OID AAC | -0.379 (-0.492 – -0.264) | -0.139 (-0.176 – -0.103) | <0.001 |
| Presence of lacunes at follow-up | 9 (31.0) | 35 (17.9) | 0.095 |
| WMH volume AAC | 1.166 (0.471 – 1.861) | 0.372 (0.266 – 0.478) | 0.005 |
| Global PVS count AAC | 2.023 (1.220 – 2.827) | 2.036 (1.779 – 2.293) | 0.317 |
| Lateral ventricular volume AAC | 1.800 (1.342 – 2.257) | 0.987 (0.889 – 1.085) | <0.001 |
| Hippocampal volume AAC | -0.141 (-0.168 – -0.115) | -0.064 (-0.073 – -0.056) | <0.001 |
| Amygdalar volume AAC | -0.033 (-0.048 – -0.018) | -0.012 (-0.017 – -0.007) | 0.003 |
| Total GM volume AAC | -4.863 (-6.333 – -3.392) | -3.570 (-3.967 – -3.173) | 0.095 |

**Supplementary Table 7** Progression of odor identification and longitudinal sMRI baseline by presence of anosmia and progression to dementia or PD

Data are AACs (95% CI) obtained from linear mixed models adjusted for demographic factors (sex, age, and education) for continuous variables, and (%) for categorical variables. *AAC* annual average change, *CI* confidence interval, *GM* gray matter, *OID* odor identification, *PVS* perivascular spaces, *WMH* white matter hyperintensities.

**Supplementary Table 8** Associations of baseline and progression of sMRI markers with annual average change in odor identification after excluding anosmic participants

|  | | *Model 1* | | | *Model 2* | | *Model 3* | |
| --- | --- | --- | --- | --- | --- | --- | --- | --- |
| sMRI markers | | β-coefficient (95% CI),  AAC in OID score | *p* | *p_FWE_* | β-coefficient (95% CI),  AAC in OID score | *p* | β-coefficient (95% CI),  AAC in OID score | *p* |
| *Baseline*^a^ | |  |  |  |  |  |  |  |
|  | Lacunes presence | -0.078 (-0.175 – 0.019) | 0.113 | 0.226 | -0.020 (-0.126 – 0.087) | 0.714 | -0.021 (-0.128 – 0.086) | 0.703 |
|  | WMH volume | -0.037 (-0.075 – 0.000) | 0.053 | 0.159 | 0.003 (-0.043 – 0.048) | 0.909 | 0.003 (-0.043 – 0.048) | 0.903 |
|  | Global PVS count | -0.043 (-0.077 – -0.009) | 0.014 | 0.070 | -0.036 (-0.072 – 0.001) | 0.054 | -0.036 (-0.072 – 0.001) | 0.054 |
|  | Lateral ventricular volume | -0.042 (-0.081 – -0.003) | 0.034 | 0.136 | -0.023 (-0.068 – 0.021) | 0.300 | -0.024 (-0.068 – 0.021) | 0.294 |
|  | Hippocampal volume | **0.054 (0.017 – 0.092)** | **0.005** | **0.030** | 0.033 (-0.020 – 0.085) | 0.221 | 0.032 (-0.020 – 0.085) | 0.229 |
|  | Amygdalar volume | 0.022 (-0.016 **–** 0.060) | 0.248 | 0.248 | -0.015 (-0.061 – 0.031) | 0.520 | -0.015 (-0.061 – 0.031) | 0.530 |
|  | Total GM volume | **0.058 (0.021 – 0.095)** | **0.002** | **0.014** | 0.034 (-0.012 – 0.081) | 0.148 | 0.034 (-0.012 – 0.080) | 0.152 |
| *Progression*^b^ | |  |  |  |  |  |  |  |
|  | Lacunes presence at follow-up | **-0.127 (-0.239 – -0.015)** | **0.027** | **0.030** | -0.073 (-0.181 – 0.034) | 0.181 | -0.074 (-0.181 – 0.034) | 0.179 |
|  | WMH volume AAC | **-1.534 (-2.492 – -0.580)** | **0.002** | **0.010** | -0.482 (-1.520 – 0.556) | 0.363 | -0.489 (-1.528 – 0.550) | 0.356 |
|  | Global PVS count AAC | **-2.130 (-3.839 – -0.421)** | **0.015** | **0.030** | **-1.808 (-3.444 – -0.172)** | **0.030** | **-1.802 (-3.439 – -0.165)** | **0.031** |
|  | Lateral ventricular volume AAC | **-1.523 (-2.593 – -0.453)** | **0.005** | **0.015** | -0.455 (-1.642 – 0.732) | 0.453 | -0.464 (-1.652 – 0.724) | 0.444 |
|  | Hippocampal volume AAC | **1.775 (0.932 – 2.618)** | **<0.001** | **<0.001** | 0.940 (-0.026 – 1.907) | 0.056 | 0.945 (-0.022 – 1.913) | 0.056 |
|  | Amygdalar volume AAC | **2.833 (1.001 – 4.666)** | **0.002** | **0.010** | 1.074 (-0.860 – 3.008) | 0.276 | 1.053 (-0.883 – 2.989) | 0.286 |
|  | Total GM volumes AAC | **2.061 (0.857 – 3.265)** | **0.001** | **0.006** | **1.296 (0.099 – 2.492)** | **0.034** | **1.286 (0.089 – 2.484)** | **0.035** |

*CI* confidence interval, *GM* gray matter, *sMRI* structural magnetic resonance imaging, *OID* odor identification, *PVS* perivascular spaces, *WMH* white matter hyperintensities.

*Model 1*, adjusted for demographic factors (sex, age, and education) and baseline sMRI markers for analyses of progression.

*Model 2*, all sMRI markers were added simultaneously to model 1.

*Model 3*, model 2 adjusted for extra covariates (smoking status and semantic memory performance).

^a^ All sMRI markers were z-transformed except lacunes (i.e., presence or absence).

^b^ AACs were computed using z-transformed sMRI markers.

Significant results in bold.

**Supplementary Table 9** Participant characteristics at baseline by progression to dementia or PD and availability of MRI markers at follow-up

|  | | | Progression to dementia or PD at follow-up | | | |  | |  | | Follow-up sMRI markers in participants without dementia or PD | | | | | |
| --- | --- | --- | --- | --- | --- | --- | --- | --- | --- | --- | --- | --- | --- | --- | --- | --- |
| Characteristics | | | Yes (*n*=58) | | No (*n*=343) | | *p* | |  | | No (*n*=147) | | Yes (*n*=196) | | *p* | |
| Age (y), *M* (*SD*) | | | 77.2 (6.8) | | 69.0 (8.4) | | <0.001 | |  | | 70.2 (9.0) | | 68.1 (7.9) | | 0.025 | |
| Age groups | | |  | |  | |  | |  | |  | |  | |  | |
|  | Young-old (<78), *n* (%) | | | 23 (39.7) | | 259 (75.5) | | <0.001 | |  | | 105 (71.4) | | 154 (78.6) | | 0.128 |
|  | Old-old (≥78), *n* (%) | | 35 (60.3) | | 84 (24.5) | |  | |  | | 42 (28.6) | | 42 (21.4) | |  | |
| Female, *n* (%) | | | 41 (70.7) | | 199 (58.0) | | 0.069 | |  | | 78 (53.1) | | 121 (61.7) | | 0.107 | |
| Education (y), *M* (*SD*) | | | 11.1 (4.3) | | 13.0 (4.2) | | 0.002 | |  | | 12.4 (4.0) | | 13.4 (4.3) | | 0.025 | |
| MMSE score, *M* (*SD*) | | | 28.7 (1.2) | | 29.2 (1.0) | | 0.006 | |  | | 29.1 (1.0) | | 29.3 (0.9) | | 0.054 | |
| Hypertension, *n* (%) | | | 32 (55.2) | | 138 (40.2) | | 0.033 | |  | | 63 (42.9) | | 75 (38.3) | | 0.391 | |
| Diabetes mellitus, *n* (%) | | | 2 (3.4) | | 26 (7.6) | | 0.402 | |  | | 14 (9.5) | | 12 (6.1) | | 0.239 | |
| Current smoking, *n* (%) | | | 8 (13.8) | | 49 (14.3) | | 0.921 | |  | | 24 (16.3) | | 25 (12.8) | | 0.350 | |
| *APOE* ε4 allele | | |  | |  | |  | |  | |  | |  | |  | |
|  | | Non carrier, *n* (%) | 33 (56.9) | | 243 (70.6) | | 0.019 | |  | | 102 (69.4) | | 141 (71.9) | | 0.948 | |
|  | | Carrier, *n* (%) | 24 (41.4) | | 89 (26.2) | |  | |  | | 37 (25.2) | | 52 (26.5) | |  | |
|  | | Missing, *n* (%) | 1 (1.7) | | 11 (3.2) | |  | |  | | 8 (5.4) | | 3 (1.5) | |  | |
| OID score, *M* (*SD*) | | | 10.4 (2.9) | | 12.4 (2.4) | | <0.001 | |  | | 12.2 (2.5) | | 12.5 (2.4) | | 0.219 | |
| Semantic memory score, *M* (*SD*)^†^ | | | 21.4 (4.8) | | 24.0 (4.1) | | <0.001 | |  | | 23.4 (4.8) | | 24.4 (3.5) | | 0.041 | |
| eTIV (mL), *M* (*SD*) | | | 1444.5 (137.9) | | 1501.7 (156.3) | | 0.009 | |  | | 1510.6 (150.0) | | 1495.0 (160.9) | | 0.359 | |
| sMRI markers | | |  | |  | |  | |  | |  | |  | |  | |
|  | | Presence of lacunes, *n* (%) | 17 (29.3) | | 55 (16.0) | | 0.015 | |  | | 25 (17.0) | | 30 (15.3) | | 0.671 | |
|  | | WMH volume (mL), *M* (*SD*) | 11.9 (14.7) | | 6.2 (8.0) | | 0.005 | |  | | 7.2 (8.7) | | 5.5 (7.5) | | 0.052 | |
|  | | Global PVS count, *M* (*SD*) | 88.8 (28.3) | | 81.8 (25.6) | | 0.058 | |  | | 82.9 (27.5) | | 81.1 (24.1) | | 0.522 | |
|  | | Lateral ventricular volume (mL), *M* (*SD*) | 46.0 (17.1) | | 36.8 (15.7) | | <0.001 | |  | | 40.2 (17.4) | | 34.2 (13.8) | | <0.001 | |
|  | | Hippocampal volume (mL), *M* (*SD*) | 7.0 (0.9) | | 7.6 (0.8) | | <0.001 | |  | | 7.5 (0.8) | | 7.7 (0.7) | | 0.022 | |
|  | | Amygdalar volume (mL), *M* (*SD*) | 2.6 (0.4) | | 2.7 (0.3) | | 0.002 | |  | | 2.7 (0.3) | | 2.8 (0.3) | | 0.468 | |
|  | | Total GM volume (mL), *M* (*SD*) | 522.9 (49.3) | | 556.0 (51.3) | | <0.001 | |  | | 550.9 (53.9) | | 559.9 (49.0) | | 0.108 | |

*eTIV* estimated total intracranial volume, *GM* gray matter, *M* mean, *MMSE* Mini Mental State Examination, *OID* odor identification, *PVS* perivascular spaces, *SD* standard deviation, *sMRI* structural magnetic resonance imaging, *WMH* white matter hyperintensities.

^†^ The number of participants with missing values was 2 for the semantic memory test (i.e., SRB:1), belonging to the group of participants who did not progress to dementia during the 15-year follow-up without available sMRI markers at follow-up.

**Supplementary Table 10** Associations of baseline and progression of sMRI markers with annual average change in odor identification after excluding dementia and PD cases at follow-up

|  | | *Model 1* | | | *Model 2* | | *Model 3* | |
| --- | --- | --- | --- | --- | --- | --- | --- | --- |
| sMRI markers | | β-coefficient (95% CI),  AAC in OID score | *p* | *p_FWE_* | β-coefficient (95% CI),  AAC in OID score | *p* | β-coefficient (95% CI),  AAC in OID score | *p* |
| *Baseline*^a^ | |  |  |  |  |  |  |  |
|  | Lacunes presence | -0.023 (-0.123 – 0.076) | 0.644 | 1.000 | 0.024 (-0.089 – 0.136) | 0.681 | 0.025 (-0.089 – 0.138) | 0.670 |
|  | WMH volume | -0.022 (-0.060 – 0.017) | 0.265 | 1.000 | -0.003 (-0.050 – 0.043) | 0.879 | -0.003 (-0.050 – 0.043) | 0.885 |
|  | Global PVS count | -0.033 (-0.068 – 0.003) | 0.072 | 0.504 | -0.033 (-0.070 – 0.005) | 0.086 | -0.033 (-0.070 – 0.005) | 0.086 |
|  | Lateral ventricular volume | -0.027 (-0.068 – 0.014) | 0.201 | 1.000 | -0.019 (-0.064 – 0.027) | 0.413 | -0.020 (-0.065 – 0.026) | 0.398 |
|  | Hippocampal volume | 0.033 (-0.008 – 0.073) | 0.114 | 0.684 | 0.025 (-0.027 – 0.078) | 0.347 | 0.024 (-0.029 – 0.077) | 0.383 |
|  | Amygdalar volume | 0.010 (-0.030 – 0.049) | 0.634 | 1.000 | -0.008 (-0.054 – 0.038) | 0.733 | -0.007 (-0.053 – 0.039) | 0.763 |
|  | Total GM volume | 0.024 (-0.013 – 0.062) | 0.206 | 1.000 | 0.007 (-0.040 – 0.053) | 0.779 | 0.007 (-0.040 – 0.054) | 0.763 |
| *Progression*^b^ | |  |  |  |  |  |  |  |
|  | Lacunes presence at follow-up | -0.095 (-0.209 – 0.019) | 0.102 | 0.102 | -0.057 (-0.167 – 0.053) | 0.313 | -0.055 (-0.165 – 0.055) | 0.324 |
|  | WMH volume AAC | **-1.278 (-2.229** – **-0.** **327)** | **0.008** | **0.040** | -0.332 (-1.368 – 0.704) | 0.530 | -0.333 (-1.369 – 0.703) | 0.529 |
|  | Global PVS count AAC | -2.071 (-3.767 – -0.376) | 0.017 | 0.051 | **-1.706 (-3.351** – **-0.061)** | **0.042** | **-1.697 (-3.342** – **-0.051)** | **0.043** |
|  | Lateral ventricular volume AAC | **-1.626 (-2.751** – **-0.500)** | **0.005** | **0.030** | -0.698 (-1.943 – 0.548) | 0.272 | -0.686 (-1.932 – 0.561) | 0.281 |
|  | Hippocampal volume AAC | **1.563 (0.662** – **2.464)** | **0.001** | **0.007** | 0.855 (-0.139 – 1.848) | 0.092 | 0.866 (-0.128 – 1.860) | 0.088 |
|  | Amygdalar volume AAC | 2.101 (0.229 – 3.973) | 0.028 | 0.056 | 0.679 (-1.261 – 2.619) | 0.493 | 0.679 (-1.261 – 2.620) | 0.492 |
|  | Total GM volume AAC | **1.645 (0.383** – **2.907)** | **0.011** | **0.044** | 0.941 (-0.317 – 2.198) | 0.143 | 0.946 (-0.312 – 2.204) | 0.141 |

*CI* confidence interval, *GM* gray matter, *sMRI* structural magnetic resonance imaging, *OID* odor identification, *PVS* perivascular spaces, *WMH* white matter hyperintensities.

*Model 1*, adjusted for demographic factors (sex, age, and education) and baseline sMRI markers for analyses of progression.

*Model 2*, all sMRI markers were added simultaneously to model 1.

*Model 3*, model 2 adjusted for extra covariates (smoking status and semantic memory performance).

^a^ All sMRI markers were z-transformed except lacunes (i.e., presence or absence).

^b^ AACs were computed using z-transformed sMRI markers.

Significant results in bold.
